# Intercellular Adhesion Molecule 3: Structure, Cellular Functions, and Emerging Role in Human Diseases

**DOI:** 10.7150/jca.100612

**Published:** 2025-01-27

**Authors:** Xinzhuang Shen, Dehong Luo, Xiaowen Yang, Yifei Li, Fuming Lian, Huan Liu, Xiaoyuan Zhang, Wenzhi Shen

**Affiliations:** 1 College of Clinical Medicine, Jining Medical University, Jining 272067, China.; 2 Shandong Provincial Precision Medicine Laboratory for Chronic Non-communicable Diseases, Institute of Precision Medicine, Jining Medical University, Jining 272067, China.; 3 The Third Affiliated Hospital of Zunyi Medical University (The First People's Hospital of Zunyi), Zunyi 563000, China.

**Keywords:** Intercellular adhesion molecule, ICAM3, LFA-1, Structure, Immune regulation, Cancer, Human disease

## Abstract

The intercellular adhesion molecule 3 (ICAM3), also known as CD50, is a member of the intercellular adhesion molecule (ICAM) family. All ICAM proteins are type I transmembrane glycoproteins containing 2-9 immunoglobulin-like C2-type structural domains and bind to the lymphocyte function-associated antigen-1 (LFA-1) protein. ICAM3 is abundantly and constitutively expressed in all leukocytes and is probably the most important ligand for LFA-1 in initiating immune responses. In recent years, more and more studies have focused on ICAM3 and found that it is closely related to the pathogenesis of various diseases. Here, we summarize the genomic localization, protein structure, and basic functions of ICAM3, and discuss the research progress of ICAM3 in mediating immune cell function and other diseases. Further, we describe the regulatory role of ICAM3 on the progression of different types of malignant cancers and the associated signaling pathways. Our work assesses the feasibility of ICAM3 as a molecular marker for the diagnosis of human diseases and cancers, which may provide new targets for treating related diseases and cancers. As a typical transmembrane protein, we expect to find or synthesize specific small molecule inhibitors for the treatment of clinically relevant diseases.

## Introduction

The intercellular adhesion molecule (ICAM) family consists of cell membrane surface glycoproteins characterized by polypeptide homologous sequences and conserved β2 integrin binding domains 1. Structurally, all ICAMs are transmembrane glycoproteins consisting of an extracellular region with an amino-terminal end, a transmembrane region, and an intracellular region with a C-terminal end. Despite their shared structural similarity and integrin-binding capabilities, members of the ICAM family differ in their expression across cell types, gene regulation patterns, and downstream signaling effector molecules. While ICAMs are known to transmit signals from the extracellular to the intracellular environment, the exact molecular mechanism is not clear.

The ICAM family is currently reported to have five molecules: ICAM1, ICAM2, ICAM3, ICAM4 and ICAM5. Among these, ICAM3 is a crucial member, extensively studied and reported for its expression and function in both normal cells, such as in different lymphocytes, and in cancer cells. The role of ICAM3 in different human diseases, particularly in cancers, has been investigated. However, there are limited systematic reviews on its functional and mechanistic characterization, and clinical applications.

In this review, we provide an overview of recent reports on ICAM3, summarizing its history, structure, and roles in human diseases. Additionally, we discuss the applications of ICAM3 in different types of cancers, providing insights into the future development of novel targeted drugs and related cancer therapies.

## 1. Discovery and structure of ICAM3

**Figure 1:**
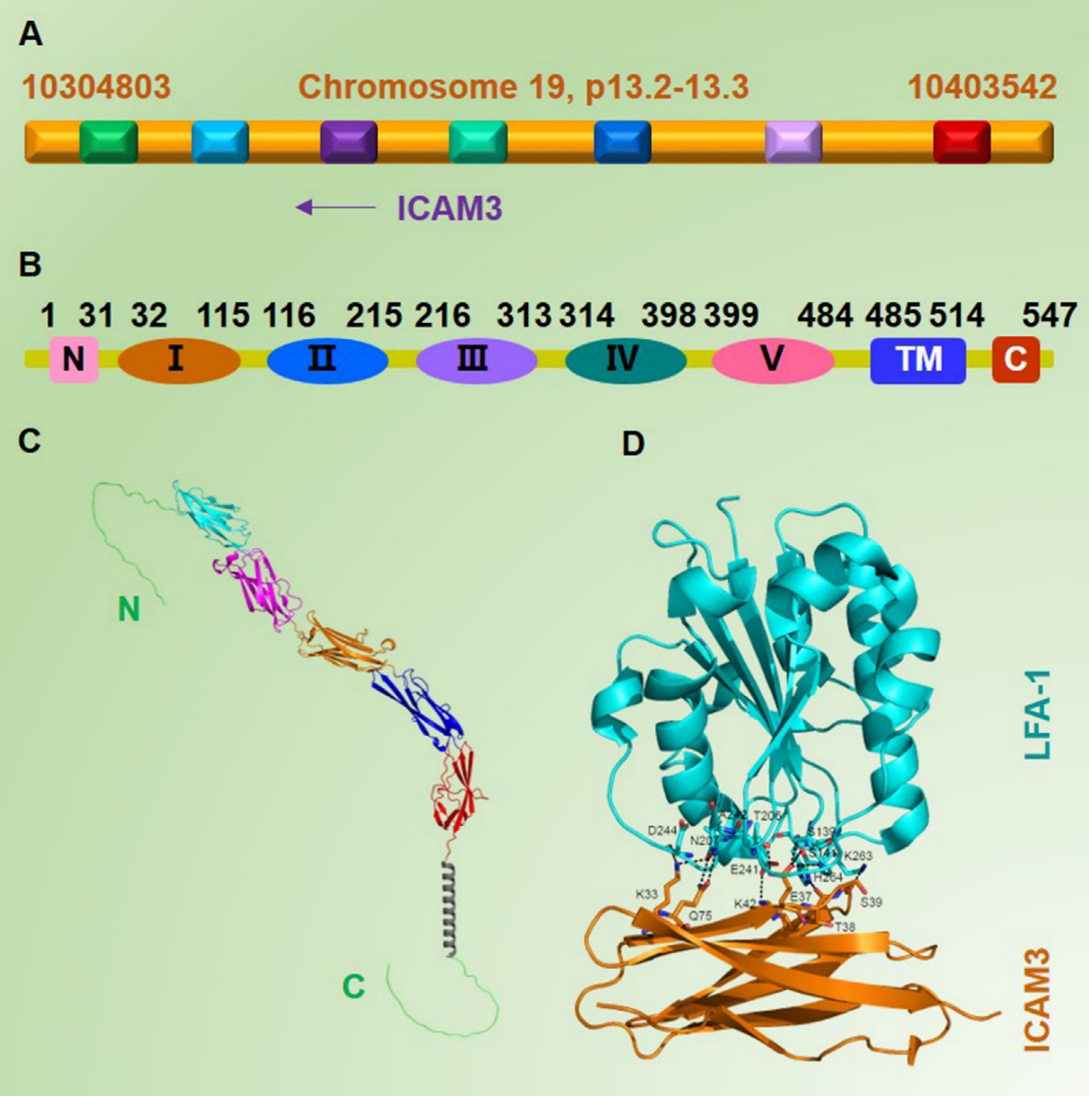
** Basic information and structure of ICAM3.** A. Location of the ICAM3 gene on the human chromosome (NCBI database). B. Information on different structural domains of ICAM3 protein (Uniprot database). C. Structure of ICAM3. D. Information on binding of ICAM3 to its receptor LFA-1 (Uniprot database). Cyan: 32-115, C2 Ig-like repeat 1. Magenta: 116-215, C2 Ig-like repeat 2. Organe: 216-313, C2 Ig-like repeat 3. Blue: 314-398, C2 Ig-like repeat 4. Red: 399-484, C2 Ig-like repeat 5. Grey (helix): 485-514, trans-membrane (TM). D. ICAM3 protein interacts with the receptor LFA-1. The residues involved in the interface between ICAM3 and LFA were shown in sticks. Dotted lines indicated the hydrogen bonds. This figure was derived from the crystal structure of ICAM3 complexd with LFA (PDB ID: 1t0p).

ICAM3 was first discovered and cloned in 1992 2-4. The human ICAM3 gene is located in the 19p13.2-p13.3 region, which is close to the ICAM1 gene (**Figure 1A**). Before its discovery, it was known that both ICAM family members (ICAM1 and ICAM2) could bind to their receptor lymphocyte function-associated antigen-1 (LFA-1) 5. Vazeux R *et al.* 6 observed that the activity of LFA-1 in mature T cells was partially inhibited by antibody blockade of ICAM1 and ICAM2, suggesting the existence of similar molecules. They also discovered a new molecule of molecular weight of about 124 kDa, which is similar to ICAM1 and ICAM2 and was named ICAM3 6. Fougerolles AR *et al.* 3 successively amplified a cDNA fragment of ICAM3, predicted its mature protein sequence, and compared it with the known protein sequences of ICAM1 and ICAM2. They found that the probable protein sequence of ICAM3 shared 51% structural similarity with ICAM1 and 37% similarity with ICAM2, suggesting a functional resemblance to ICAM1, though further experiments were needed to confirm this. Further analysis and validation revealed that ICAM3, also known as CD50 7, has a total gene length of 1644 bp, translating to 547 amino acids. Like other ICAMs, ICAM3 is a typical type I transmembrane glycoprotein, and its extracellular structural domain (N-terminal structural domain) contains five type C2 Ig-like repeats consisting of 484 amino acids; a transmembrane segment (transmembrane structural domain) of 30 amino acids, and an intracellular segment (C-terminal structural domain) of 34 amino acids (**Figure 1B & 1C**). As an adhesion molecule, ICAM3 is mainly localized on the cell membrane.

More specifically, unlike ICAM1 and ICAM2, the ICAM3 gene is absent in rodents, and it is possible that ICAM3 was lost during mammalian evolution due to gene deletions 8. This situation is one of the major limiting factors in studying the pathophysiological functions of ICAM3. Due to the lack of ICAM3 genetically engineered mice that could help to explore the function of ICAM3 throughout the organism, all studies on whether the physiological functions of ICAM3 might be replaced by ICAM1 and ICAM2 or whether they are unique and irreplaceable have been inconclusive. On the other hand, this uniqueness of ICAM3 could be considered as an opportunity to study the cell adhesion mechanisms specifically involved in human immunity 9.

## 2. Binding analysis of ICAM3 and LFA-1

Several ICAM3 functions are mediated by binding to LFA-1, and many studies have focused on investigating the binding relationship between LFA-1 as a co-receptor for ICAM1, ICAM2, and ICAM3. Binnerts ME *et al.* 10 found that while the binding sites on LFA-1 for ICAM1, ICAM2, and ICAM3 are conserved, those on ICAM1, ICAM2, and ICAM3, to LFA-1 are different. The authors found that the binding site of ICAM3 to LFA-1 is conserved in addition to the non-contiguous amino acids, which provides a basis for further investigation of the function and mechanism of ICAM3.

Early studies reported that ICAM3, as the most important ligand of LFA-1, plays an important role in initiating the immune response through receptor-ligand binding. They found that ICAM1, ICAM2, and ICAM3 all provide important signals for immune cell adhesion during cell interactions. ICAM1 expression can be induced by cytokines in the presence of inflammation and injury, whereas ICAM3, which functions similarly to ICAM1 in this regard, is also inducible by inflammatory cytokines, providing important signals for lymphocyte trapping. The authors also revealed that ICAM3 binds to LFA-1 through two immunoglobulin domains at its extracellular domain 3.

Numerous studies have shown that although the affinity of ICAM3 for LFA-1 is 9-fold lower than that of ICAM1 for LFA-1, the interaction of ICAM3/LFA-1 is thought to play a major role in the early stages of the adaptive immune response when naïve T lymphocytes establish their first contact with antigen-presenting cells (APCs) due to the near-absence of ICAM-1 on resting T cells and the very low level of ICAM2 on them as compared to ICAM-3 10-12. Therefore, at the early stage of the adaptive immune response, when naïve T lymphocytes establish their first contact with antigens presented by antigen-presenting cells (APCs), the ICAM3/LFA-1 interaction is thought to play a major role 9 (**Figure 1D**).

## 3. Function of ICAM3 in immune cells

### 3. 1 Role of ICAM3 in leukocytes

Fawcett J *et al.* 2 constructed an *in vitro* plasmid expression vector for ICAM3 and ectopically expressed it in leukocytes. They found that ICAM3 functioned similarly to ICAM1 in resting leukocytes but served as the primary ligand for the co-receptor LFA-1, playing an essential role in the immune response.

### 3.2 Role of ICAM3 in T cells

Juan M *et al.* 13, 14 performed functional assays of ICAM3 in T cells and found that the expression of ICAM3 varies at different stages of T cell development. This expression gradually increases with the progressive development of T cells, then peaks in memory T cells, indicating that the expression of ICAM3 might be closely related to the development of T cells. Also, the exogenous transfer of ICAM3 into T cells results in a transient upward fluctuation of Ca^2+^ levels in T cells, though the mechanism remains unclear.

It has also been reported that the binding of ICAM3 to LFA-1 plays an important role in mediating T-cell motility or migration, as ICAM3 is reported to alter the migration process of T cells, mainly through competitive binding of the common ligand LFA-1 with ICAM1, thus exerting significant regulatory effect on the ICAM1-LFA-1 pathway 15.

Subsequent studies have further revealed the function of ICAM3. Arroyo AG *et al.* 16 found that during T-cell activation, ICAM3 binds to the receptor LFA-1, which mediates the activation of tyrosine kinases and promotes the phosphorylation of tyrosine proteins to activate the downstream pathway. The CD45 tyrosine phosphatase also plays an important role in the process, but the specific mechanism is unclear 16 17. This study clarifies the direction for further exploration of the function of ICAM3. Based on above research, Skubitz KM *et al.* providing strong evidence that tyrosine sites in the intracellular segment of ICAM3 can be phosphorylated by tyrosine kinases, which in turn promotes the activation of these kinases. However, the authors did not specify the kinases or their molecular mechanisms 18.

### 3.3 Other functions mediated by ICAM3

**Figure 2:**
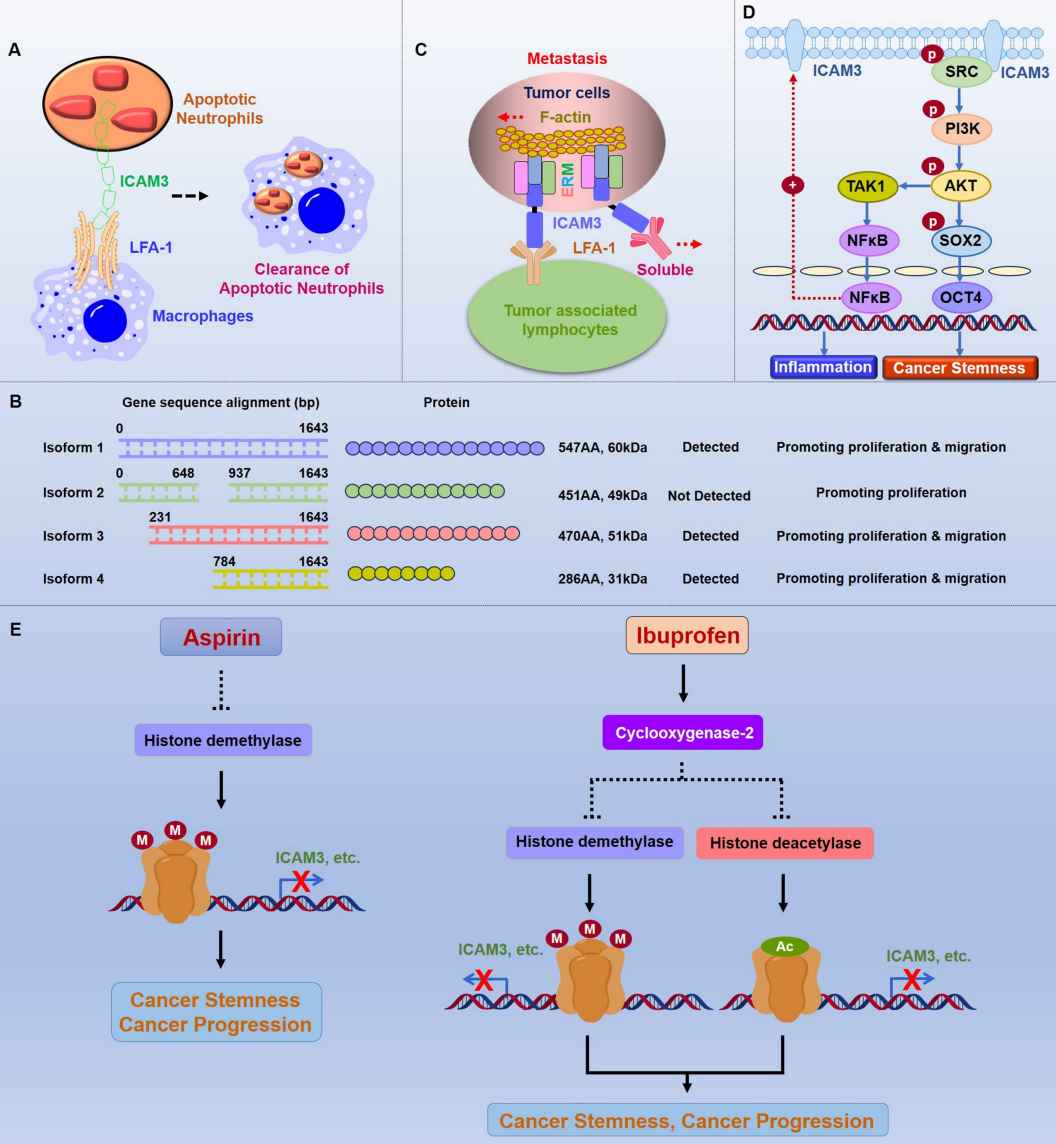
** Function and mechanism of ICAM3 in cancers.** A. Mechanisms of ICAM3 in apoptotic neutrophil clearance. B. Different ICAM3 transcript variants have distinct functions in DLBCL. C. Function and mechanism of ICAM3 in breast and lung cancer metastasis. D. Function and mechanism of ICAM3 in mediating inflammation and cancer cell stemness. E. Role of ICAM3 in aspirin- and ibuprofen-mediated inhibition of cancer progression.

The function of ICAM3 has been extended to other cell types. Torr EE *et al.* found that ICAM3 binding to LFA-1 plays an important role in the removal of apoptotic neutrophils. After phagocytosing cellular foreign bodies, neutrophils gradually move towards apoptosis, and apoptotic neutrophils are cleared by macrophages through the combination of ICAM3, which is highly expressed on the neutrophil cell surface, and LFA-1, which is expressed on the surface of macrophages (**Figure 2A**) 19.

Estecha A *et al.* 20 further found that the promoter region of ICAM3 can be bound by co-transcription factors such as ETs and C/EBP, promoting macrophage maturation as well as monocyte extravasation. More reports on ICAM3 have demonstrated that ICAM3 is widely expressed not only in lymphocytes such as T and B cells but also in many endothelial cells and dendritic cells 21-27. Although there have been many reports of ICAM3 in normal cells, specific mechanisms remain unclear and require further exploration and validation.

## 4. Function of ICAM3 in cancers

### 4.1 ICAM3 and B-cell lymphoma

B-cell lymphomas are solid tumors arising from B cells, including Hodgkin's and non-Hodgkin's lymphoma. Molica S *et al.* demonstrated in successive articles on leukemias that in B-cell chronic lymphomas, ICAM3 also exhibits a high state of expression, further demonstrating its potential as a cancer-associated marker 28. Furthermore, Darom A *et al.* analyzed 36 cases of B-cell MALT-type primary gastric lymphomas and identified ICAM3, along with PECAM-1 and HLA-DR, as markers of tumor expansion potential and host immune surveillance, respectively. These findings suggested that using these markers in combination may help us identify high-risk patients who may benefit from more aggressive treatment regimens 29. Li L *et al.* examined the sequence and expression of ICAM3 transcript variants 1-4 in diffuse large B-cell lymphoma (DLBCL) cells and tumor tissues, and showed that the variant 1 is the longest variant which has 1644 base pair (bp) and correspondingly 547 Amino Acid (AA), the molecule weight is 60kDa. The variant 2 has 1356 bp (less 288 bp in the middle part compare to variant 1) and correspondingly 451 Amino Acid (AA), the molecule weight is 49kDa. The variant 3 has 1413 bp (less 231 bp in the beginning part compare to variant 1) and correspondingly 470 Amino Acid (AA), the molecule weight is 51kDa. The variant 4 has 861 bp (less 783 bp in the beginning part compare to variant 1) and correspondingly 286 Amino Acid (AA), the molecule weight is 31kDa. Moreover, variants1, 3, and 4 were expressed in normal B-cell lines, in three DLBCL cell lines (except SU-DHL-2) and tumor tissues, while no expression of variant 2 was detected. Variants 1-4 accelerated the cell cycle and enhanced cell proliferation in SU-DHL-2 cells in vitro. However, overexpression of variants 1-4 had no effect on apoptosis in SU-DHL-2 cells. Expression of variants 1, 3, and 4 also promoted cell migration and epithelial-mesenchymal transition process, while variant 2 had no effect (**Figure 2B**). These findings suggest that different ICAM3 transcript variants have distinct functions in DLBCL 30.

### 4.2 ICAM3 and Breast Cancer (BC)

BC is the most prevalent malignant tumor globally and the leading cause of cancer deaths in women. Early studies by Fox SB *et al.* found that ICAM3 is highly expressed in breast cancer cells, suggesting that ICAM3 might be a marker for breast cancer 31. In another study, using the triple-negative breast cancer (TNBC) cell line IIB-BR-G as a model, researchers explored the changes in protein profiling expression in lung metastasis secondary to breast cancer. They found that ICAM3 expression is downregulated in the low-proliferative and high-metastatic cell line IIB-BR-G-MTS6, indicating a possible link between ICAM3 and the proliferation and metastasis of TNBC cells 32. Alonso EN *et al.* demonstrated by RNA sequencing that maitake D-fraction fiber altered the expression of several genes involved in the stimulation of apoptosis, such as ICAM3, which may lead to inhibition of cell growth and proliferation, cell cycle arrest, migration and metastasis blockade, and dose-dependent induction of multidrug sensitivity 33. Shen *et al.*. found that ICAM3 expression is closely associated with TNM staging in human breast and lung cancers, and is dominantly overexpressed in highly invasive cancer cell lines (231 and A549 cells). Furthermore, *in vitro* and *in vivo*, knockdown of ICAM3 inhibits cancer metastasis, whereas ectopic expression of ICAM3 promotes cancer metastasis. Exploration of the underlying mechanisms suggests that ICAM3 not only binds to LFA-1 through its extracellular structural domain and structural protein ERM but also binds to lamellipodia through its intracellular structural domain, thereby generating tensile forces that facilitate metastasis (**Figure 2C**) 34.

### 4.3 ICAM3 and lung cancer

Lung cancer has a high incidence and mortality rate worldwide 35. Kim YG *et al.* 36 reported that ICAM3 promotes the proliferation of non-small cell lung cancer (NSCLC) cell line H1299 and speculated that the process might be through the PI3K-AKT signaling pathway, though the exact mechanism remains unclear. They also found that ICAM3 promotes the migration process of NSCLC cell line H1299 cells, potentially by affecting MMPase expression and activity, but the specific mechanism is not well elaborated 37. The same group has investigated the migration process of the MMP enzyme, and also examined the effect of ICAM3 on apoptosis in lung cancer cell line H1299, discovering that ICAM3 was able to inhibit the apoptotic process of H1299 cells, possibly through the activation of the AKT-CREB pathway 38. In 2023, Shu *et al.* used machine learning to identify key genes associated with tumor-infiltrating plasma cells (PCs) in lung adenocarcinoma patients. A prognostic model called PC score was developed using TCGA data and validated with the GEO cohort. The latest relevant results showed that 17 genes, including ICAM3, are associated with PC 39.

### 4.4 ICAM3 and multiple myeloma

Multiple myeloma is accompanied by alterations in the normal PC proteome, which can lead to changes in the tumor microenvironment and affect disease progression. Setayesh SM *et al.* 40 analyzed approximately 87,000 cells from seven patient samples (bone marrow and peripheral blood) from the myeloma disease spectrum using a single-cell high-definition liquid biopsy assay and imaging mass spectrometry and characterized the expression of clinical markers used for PC classification, other potential therapeutic targets, and cells from the tumor microenvironment, using a multiplexed panel. The analysis showed that BCMA, ICAM3 (CD50), CD221, and CS1 (SLAMF7) are the most abundantly expressed markers at all stages of myeloma, with BCMA, ICAM3, and CD221 being expressed at significantly higher levels in diseased rather than precursor PCs.

### 4.5 ICAM3 and cervical cancer

To find markers that would predict the efficacy of radiation therapy for human cervical cancer, Chung YM *et al.* 41 compared the gene expression profiles of parental SiHa cervical cancer cells and radiation-resistant SiHa/R cells using microarray technology. Microarray and northern blot analyses showed upregulation of ICAM3 expression in SiHa/R, which enhanced their resistance to radiotherapy. Further data analysis of the case samples concluded that ICAM3 was associated with radiotherapy resistance in cervical cancer. It was concluded that the expression of ICAM3 could be a valuable biomarker to predict the radiation resistance of cervical cancer during radiotherapy.

### 4.6 ICAM3 and cancer stem cells

Existing research suggests that cancer stem cells play a key role as "seed" cells in the initiation and progression of different types of colorectal cancers. Shen W *et al.* constructed a siRNA library of 1027 inflammatory genes using breast cancer stem cells as a model, with a luciferase system linked to the OCT4 promoter as a tool for high-throughput screening of inflammatory genes to regulate cancer stemness. They found that the knockdown expression of the candidate gene ICAM3 significantly inhibits the promoter activity of OCT4 and suppresses the proportion of ALDH^+^ cancer stem cells. Further functional validation and mechanistic studies revealed that ICAM3 is highly expressed in various types of cancers, such as breast cancer and lung cancer, compared to normal tissues and that ICAM3 activates Src through the intracellular segment YLPL sequence recruitment, which in turn activates the PI3K/AKT signaling pathway, enhancing the activity of the stemness molecule OCT4 and mediating cancer stemness. Additionally, ICAM3 promotes NF-κB nucleation through the Src/PI3K/AKT pathway, which then binds to the ICAM3 promoter, promoting ICAM3 expression while mediating the secretion of inflammatory factors. Small-molecule inhibitors targeting ICAM3 signaling molecules, such as Src and PI3K, could markedly inhibit ICAM3 expression, inflammation, and cancer stem cell stemness, suggesting a close relationship between ICAM3 and cancer stem cells (**Figure 2D**) 42.

### 4.7 ICAM3 is involved in anti-inflammatory drugs against cancers

In previous studies of dry eye disease, Lifitegrast, as an innovative product targeting the mechanism of ocular inflammation 43, was able to inhibit the binding of LFA-1 to ICAM-1, thereby reducing the level of inflammation mediated by T-lymphocytes, and not only treating the eye damage caused by dry eye disease, but also relieving the discomfort brought about 44. In the cancer cell *in vitro* studies, Shen W *et al.* found that Lifitegrast is also more able to reduce the binding of ICAM3 and LFA-1, inhibit the migration and movement of cancer cells caused by the binding of ICAM3/LFA-1, and then inhibit the cancer metastasis and cancer progression, which provides a new basis for the treatment of metastatic cancers 34.

Shen W *et al.* 45, 46 (**Figure 2E**) discovered that aspirin inhibits the expression of inflammation-associated stemness genes, particularly ICAM3, identified by high-throughput siRNA platforms. The underlying mechanism involves aspirin reducing lysine demethylase 6A/B (KDM6A/B) expression, which mediates histone methylation and inhibits gene expression in a COX-independent manner. Similarly, ibuprofen inhibits the expression of inflammation-associated stemness genes, particularly ICAM3, by reducing the expression of histone deacetylase (HDAC) and KDM6A/B, which mediate histone acetylation and methylation, respectively, and inhibit gene expression in a COX2-dependent manner. Therapeutically, aspirin combined with HDM inhibitors, ICAM3 downstream signaling Src/PI3K inhibitors, or ICAM3/LFA-1 inhibitors (Lifitegrast), as well as ibuprofen combined with HDAC/HDM inhibitors, prevent cancer progression *in vivo* 45, 46.

### 4.8 Expression and survival correlation analysis of ICAM3 in various cancers

**Figure 3:**
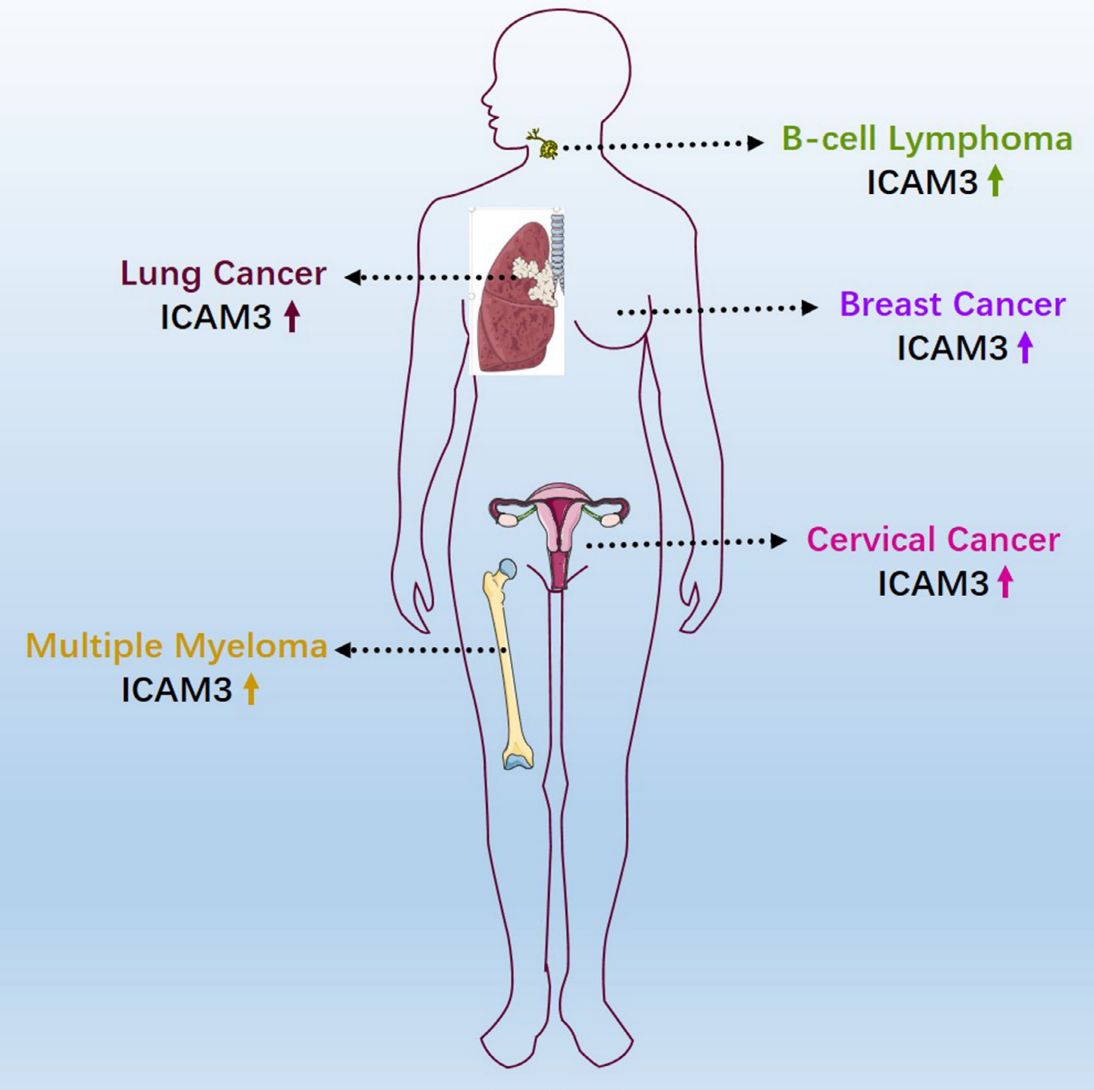
Reported expression and role of ICAM3 in different cancers.

**Figure 4:**
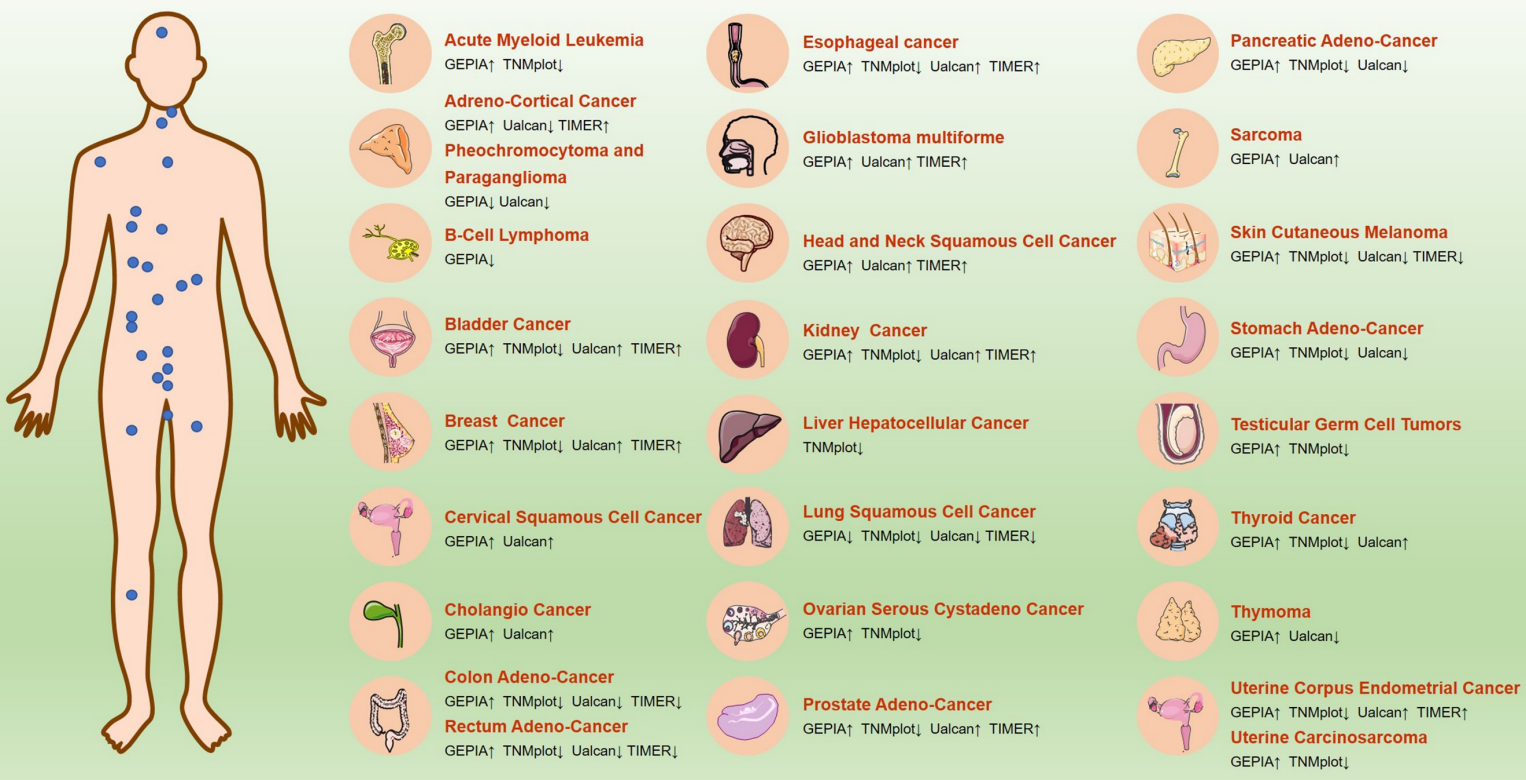
ICAM3 expression in different cancers in GEPIA, TNMplot, UALCAN and TIMER online databases.

ICAM3 may have different expressions and functions across various cancer types. We first summarized the expression of ICAM3 in cancer types that have been reported and studied (**Figure 3**). Further, we summarized ICAM3 expression in 31 cancers using the GEPIA, TIMER, UALCAN and TNMplot databases (**Figure 4**). However, the unexpected result was that ICAM3 expression was not the same in the same cancer type, in different databases. For example, in acute granulocytic leukemia, relative to normal, GEPIA showed high expression of ICAM3, while TNMplot showed low expression; in renal cancer, relative to normal, GEPIA and TIMER showed high expression, while TNMplot and UALCAN showed low expression. Possible reasons for this phenomenon include 1) cancer sample sources from different regions or races, 2) different sample sizes for normal or cancer, 3) different detection and data processing methods, etc. Nonetheless, the above data can provide suggestions and basis for subsequent studies on the expression and role of ICAM3 in different cancers.

Based on the GEPIA and UALCAN databases, we also summarized the correlation between ICAM3 expression and cancer patient survival (**Table 1**), which describes the impact of ICAM3 expression on cancer patient survival in different cancer types, and may provide a basis for subsequent studies of ICAM3 in cancer survival and prognosis.

## 5. Discussion: Relevant roles of ICAM3 in human diseases

### 5.1 ICAM3 and Multiple Sclerosis

**Figure 5:**
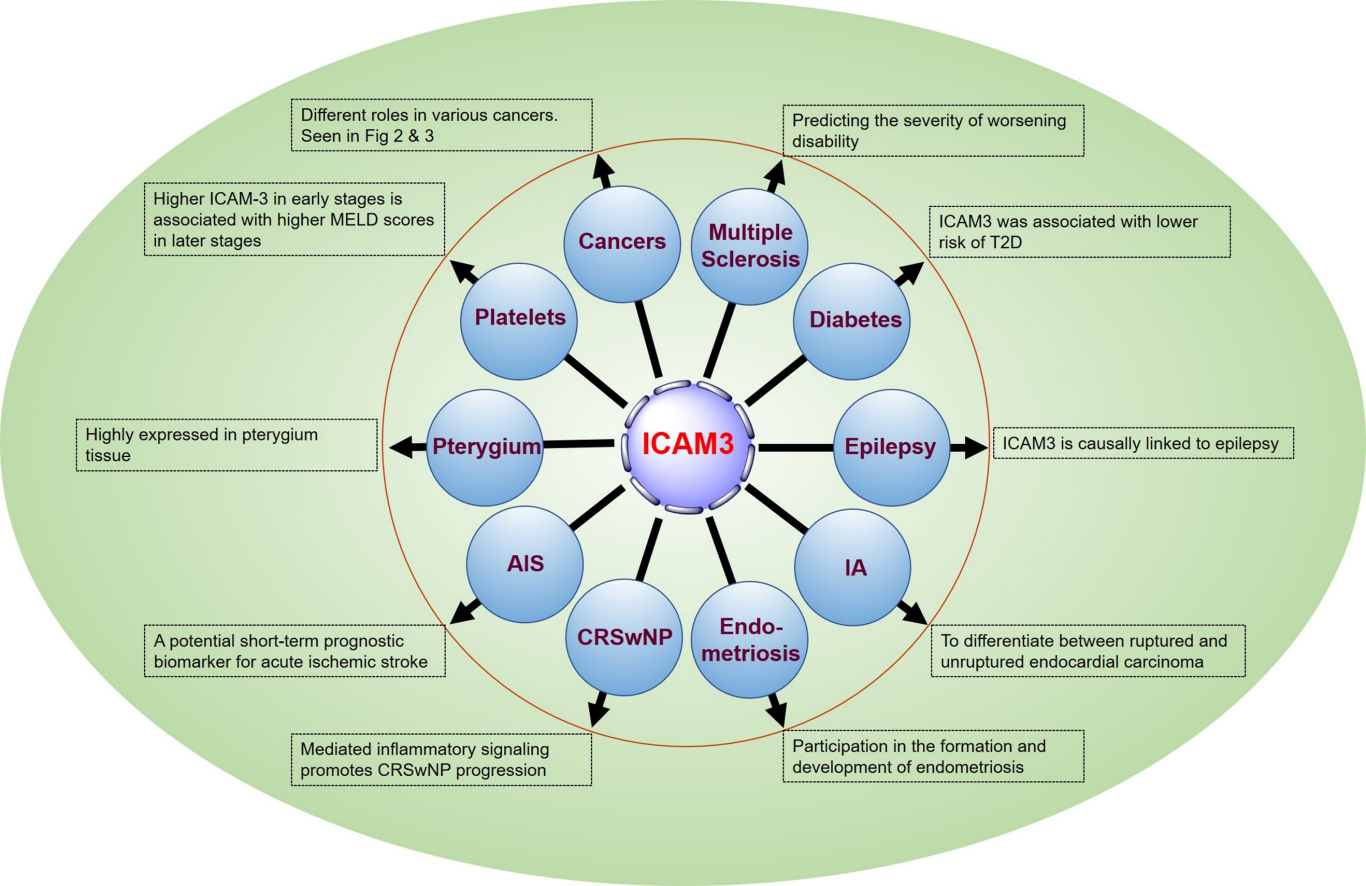
The emerging role of ICAM3 in human diseases.

Åkesson J *et al.* 47 identified 11 CSF proteins (CXCL13, LTA, FCN2, ICAM3, LY9, SLAMF7, TYMP, CHI3L1, FYB1, TNFRSF1B, and NfL), including ICAM3, through proteomics to predict the severity of disability worsening based on normalized age-related multiple sclerosis severity scores (replication AUC = 0.90) (**Figure 5**).

### 5.2 ICAM3 and vascular damage and type 2 diabetes mellitus

There is a lack of data on vascular damage biomarkers and risk of type 2 diabetes (T2D) in prospective studies. Pletsch-Borba L *et al.* performed a meta-analysis, evaluating seven biomarkers of vascular damage associated with T2D. They found that ICAM3 was associated with a lower risk of T2D through a baseline follow-up of up to 16 years (median), as well as in quantitative analysis of multiple molecules, including ICAM3, in 2,224 participants from the EPIC-Heidelberg cohort. However, further studies are needed to confirm this finding 48 (**Figure 5**).

### 5.3 ICAM3 and epilepsy

Lu M *et al.* 49 conducted brain transcriptome-wide and protein-wide association studies with chemical-gene interaction analysis identified 287 environmental chemicals and five important genes related to epilepsy (*WIPF1*, *IQSEC1, JAM2, ICAM3, and ZNF143*). ICAM3 may become a new target for drug prediction and development (**Figure 5**).

### 5.4 ICAM3 and Intracranial Aneurysms (IA)

Protein biomarkers of IA are essential for early detection and prediction of aneurysm rupture, contributing to the diagnosis and clinical management of the disease, monitoring treatment response, and detecting recurrence. Xiong Y *et al.* 50 analyzed tissues from animal models (4 cases) and sera from human patients (60 cases) using a quantitative proteomics approach based on equal-weighted tandem mass tagging to develop a comprehensive strategy for the discovery of intracranial aneurysm biomarkers. The studies identified 4811 and 562 proteins from aneurysm tissue and serum samples, respectively. Combined with logistic regression modeling, a diagnostic classifier P2 (FCN2 or RARRES2) was established to differentiate endocardial carcinomas from healthy controls with an accuracy of 93.3%, and a diagnostic classifier P7 (ADAM12, APOL3, F9, C3, CEACAM1, ICAM3, KLHDC7A) was established to differentiate ruptured endocardial carcinomas from unruptured endocardial cancer with an accuracy of 95.0%. The results of this study provide a valuable strategy for the discovery of biomarkers for IA and patient stratification (**Figure 5**).

### 5.5 ICAM3 and endometriosis

It has been reported that certain soluble cell adhesion molecules and co-proteins (sICAM-2, -3, -4, and syndecan- 1, -4) are involved in the formation and development of endometriosis. Janusz A *et al.* 51 conducted an enzyme-linked immunosorbent assay (ELISA) to determine the concentration of selected sICAMs and syndecans in the peritoneal fluid of 80 women in the proliferative phase of the menstrual cycle. The results showed a decrease in the concentration of sICAM-2 and an increase in the concentrations of sICAM-3, sICAM-4, and syndnecans-1 and -4 in the peritoneal fluid of women with endometriosis compared to the reference group (p < 0.0001). In addition, concentrations of sICAM-3 and sICAM-2 were negatively correlated in women with endometriosis. These findings suggest that the evaluated molecules play a role in regulating immune response, angiogenesis, and endometrial cell apoptosis (**Figure 5**).

### 5.6 ICAM3 and chronic sinusitis with nasal polyps

Chronic rhinosinusitis with nasal polyps (CRSwNP) is mainly characterized by eosinophils and T helper 2 cells (Th2) inflammation. Integrins and ICAMs promote immune cell recruitment and migration and have been implicated in coordinating eosinophil and Th2 cell adhesion and signaling in asthma. Blight BJ *et al.* 52 conducted a prospective observational study using peripheral blood and presigmoid tissue from CRSwNP patients (32) and controls (15). The results suggested that integrins and ICAM genes were significantly elevated in the blood (p < 0.001 to p < 0.05) and sinuses (p < 0.0003 to p < 0.05) of patients with CRSwNP compared to controls. *ITGAM, ITGAX, ITGB2,* and *ICAM3* were significantly higher in the blood (p < 0.01 to p < 0.05) and sinuses (p < 0.0003 to p < 0.05). 0.05) and genes expressed in the sinuses (p < 0.01) were strongly positively correlated. The number of ITGAM-, ITGB2-, ICAM-3- and ICAM-3/ITGB2-positive cells was significantly increased (p < 0.0001 to p < 0.04), and a positive correlation was observed between the number of ICAM3/ITGB2- and ITGAM-positive cells in CRSwNP (p < 0.02), compared to controls. These findings suggest a potential role for the integrin complexes ITGAM/ITGB2 and ICAM3 in inflammation-mediated signaling in CRSwNP (**Figure 5**).

### 5.7 ICAM3 and acute ischemic stroke

While there are studies suggesting that increased circulating ICAM3 is associated with the risk of acute ischemic stroke, the relationship between serum levels of ICAM3 and the severity and short-term prognosis of ischemic stroke is not clear. Hu Z *et al.* 53 measured baseline serum ICAM3 concentrations in 152 stroke patients with supraventricular infarction and 133 healthy controls using ELISA. The patients were followed for 2 weeks after admission to the hospital for functional prognosis, and serum ICAM3 concentrations were found to be independent of stroke severity at baseline according to the National Institutes of Health Stroke Scale and infarct volume. However, serum ICAM3 levels were positively associated with the modified Rankin Scale score at 2 weeks after hospital admission. In addition, regression analysis revealed that elevated serum ICAM3 levels were associated with a poorer short-term functional prognosis for stroke. The results of this study suggest that circulating ICAM3 may be a potential short-term prognostic biomarker for acute ischemic stroke (**Figure 5**).

### 5.8 ICAM3 and pterygium

Pterygium is one of the most common ocular surface diseases characterized by inflammatory infiltration, proliferation, angiogenesis, fibrosis, and extracellular matrix destruction. Demiryürek S *et al.* 54 detected the expression of ICAM2 and ICAM3 both on gene and protein levels in pterygium. The results showed a significant increase in ICAM2 and ICAM3 gene expression in pterygium tissues (p = 0.0018 and p = 0.0023, respectively). ICAM2 and ICAM3 protein expressions were also significantly increased in pterygium tissues (p = 0.0116 and p = 0.0252, respectively). In the immunohistochemical study, ICAM3 protein expression was significantly increased in pterygium tissue (p = 0.0152), whereas ICAM2 protein expression was not increased (p = 0. 1041). There was a significant positive correlation between pterygium grade, ICAM2 protein expression (p = 0.0398), and ICAM3 immunohistochemical score (p = 0.0138). These results demonstrate, for the first time, the expression of ICAM2 and ICAM3 in pterygium, providing insight into the signaling mechanism during pterygium formation and providing new therapeutic strategies for the treatment of pterygium (**Figure 5**).

### 5.9 ICAM3 and platelets

Thrombocytopenia has been known to be a marker of advanced liver disease. Ali RO *et al.* 55 found a negative correlation between platelets and ICAM3 in patients with hepatitis C infection presenting with early fibrosis and cirrhosis. Soluble ICAM3 was associated with increased fibrosis, liver enzymes, and synthetic dysfunction. Higher ICAM3 in early stages was associated with higher Model for End-Stage Liver Disease scores in later stages (**Figure 5**).

## 6. Conclusion and Perspective

The discovery and characterization of ICAM3 pointed out in the above findings have revealed its significant involvement in the progression of various human diseases, including cancers. However, current studies on the ICAM3 gene or protein in diseases mainly focus on phenotypic factors or bioinformatics analysis, and literature relating to the kinases directly affected as well as the specific molecular mechanisms of ICAM3 protein in the progression of related diseases are poorly understood. Therefore, more in-depth studies are needed to clarify the exact functions of ICAM3 proteins and explore their various molecular mechanisms to provide a theoretical basis for more effective disease treatment.

Additionally, as a disease associated marker, there is a lack of the detection methods for ICAM3. By clarifying the expression changes of ICAM3 in different diseases, researchers could develop novel detection methods for ICAM3 as a disease marker. For instance, ICAM3 expression varies in different cancer types, and its association with biological processes such as cancer cell proliferation, apoptosis, cancer stem cells, and prognosis suggests its potential as a cancer therapy biomarker. Developing efficient drugs for cancer treatment is crucial, and the role of ICAM3 in cancer, though not specific to different cancer types, highlights its importance. Analysis of ICAM3 expression in multiple cancers using online databases and research reports indicates that different ICAM3 subgroups have significant specificity for various cancers (**Figures 3 & 4, Table 1**), which may help in the diagnosis and treatment of such cancers. Further, the design and synthesis of small-molecule inhibitors or activators specific for ICAM3 based on the resolution of ICAM3 structure may provide new targets and therapeutic strategies for metabolic disorder diseases and cancers. To this end, it may be advisable to design molecular inhibitors of ICAM3 or explore more miRNA targets to regulate ICAM3 expression in metabolic disorders and cancers, or to combine them with small-molecule inhibitors of its affected kinases, such as PI3K/AKT or AKT/CREB.

Cancer immune cells and cancer immune microenvironment play crucial roles in malignant cancer progression, and ICAM3 is a membrane protein that can regulate immune cell function, inflammation, and cancer stem cell association. In the research for novel cancer immunotherapy strategies, ICAM3 should be considered an important target for cancer immunotherapy in the future. Consequently, treatment regimens combined with chemotherapy and other adjuvant therapies may provide strategies for the treatment of cancers or immune-related diseases.

Collectively, this review summarized the genomic localization, protein structure, and basic functions of ICAM3, and discussed the research progress of ICAM3 in mediating immune cell function and other diseases. Further described the regulatory role of ICAM3 on the progression of different types of malignant cancers and the associated signaling pathways. And assessed the feasibility of ICAM3 as a molecular marker for the diagnosis of human diseases and cancers, which may provide new targets for treating related diseases and cancers. Finally, it was expected to find or synthesize specific small molecule inhibitors for the treatment of clinically relevant human diseases.

## Figures and Tables

**Table 1:** ** Correlation between ICAM3 expression and survival of cancer patients.** Positively means that expression of ICAM3 is positively correlated with patients survival. Negatively means that expression of ICAM3 is negatively correlated with patients survival. NS means not significant.

Cancer Types	GEPIA	UALCAN
Acute Myeloid Leukemia	Negatively	Negatively
Adreno-Cortical Cancer	NS	Negatively
Pheochromocytoma and Paraganglioma	NS	NS
B-Cell Lymphoma	-	NS
Bladder Cancer	NS	NS
Breast Cancer	NS	NS
Cervical Squamous Cell Cancer	NS	Positively
Cholangio Cancer	NS	NS
Colon Adeno-Cancer	NS	NS
Rectum Adeno-Cancer	Positively	NS
Esophageal cancer	NS	Positively
Glioblastoma multiforme	NS	NS
Head and Neck Squamous Cell Cancer	Positively	NS
Kidney Cancer	NS	Negatively
Liver Hepatocellular Cancer	NS	NS
Lung Squamous Cell Cancer	NS	NS
Ovarian Serous Cystadeno Cancer	Positively	Positively
Prostate Adeno-Cancer	NS	NS
Pancreatic Adeno-Cancer	NS	NS
Sarcoma	NS	NS
Skin Cutaneous Melanoma	Positively	Positively
Stomach Adeno-Cancer	NS	-
Testicular Germ Cell Tumors	NS	NS
Thyroid Cancer	NS	NS
Thymoma	Positively	Positively
Uterine Corpus Endometrial Cancer	NS	NS
Uterine Carcinosarcoma	NS	NS

## Abbreviations

ALDH: Acetaldehyde Dehydrogenase
BC: Breast Cancer
BCMA: Targeting B Cell Maturation Antigen
CRSwNP: Chronic rhinosinusitis with nasal polyps
DLBCL: Diffuse large B-cell lymphoma
ERM: Ezrin/Radixin/Moesin
GEO: Gene Expression Omnibus
HDAC: Histone deacetylase
HDSCA: High-definition liquid biopsy assay
IA: Intracranial aneurysms
ICAM: Intercellular adhesion molecule
KDM6A/B: Lysine demethylase 6A/B
LFA-1: Lymphocyte function-associated antigen-1
NSCLC: Non-small cell lung cancer
T2D: Type 2 diabetes
TNBC: Triple-negative breast cancer
TNM: Tumor, lymph node, metastasis
TCGA: The Cancer Genome Atlas Program
